# Human granzyme B regulatory B cells prevent effector CD4+CD25- T cell proliferation through a mechanism dependent from lymphotoxin alpha

**DOI:** 10.3389/fimmu.2023.1183714

**Published:** 2023-07-31

**Authors:** Nicolas Sailliet, Hoa-Le Mai, Amandine Dupuy, Gaëlle Tilly, Cynthia Fourgeux, Martin Braud, Magali Giral, Jean-Michel Robert, Nicolas Degauque, Richard Danger, Jeremie Poschmann, Sophie Brouard

**Affiliations:** ^1^ CHU Nantes, Nantes Université, INSERM, Center for Research in Transplantation and Translational Immunology (CR2TI), UMR 1064, ITUN, Nantes, France; ^2^ Institut De Recherche En Santé 2, Cibles Et Médicaments Des Infections Et De l’Immunité IICiMed-UR1155, Nantes Université, Nantes, France

**Keywords:** regulatory B cell, regulation, single cell RNAseq, tolerance, GZMB, LTA

## Abstract

**Introduction:**

Human Granzyme B (GZMB) regulatory B cells (Bregs) have suppressive properties on CD4+ effector T cells by a mechanism partially dependent on GZMB. Moreover, these cells may be easily induced in vitro making them interesting for cell therapy.

**Methods:**

We characterized this population of in vitro induced GZMB+Bregs using single cell transcriptomics. To investigate their regulatory properties, Bregs or total B cells were also co-cultured with T cells and scRNAseq was used to identify receptor ligand interactions and to reveal gene expression changes in the T cells.

**Results:**

We find that Bregs exhibit a unique set of 149 genes differentially expressed and which are implicated in proliferation, apoptosis, metabolism, and altered antigen presentation capacity consistent with their differentiated B cells profile. Notably, Bregs induced a strong inhibition of T cell genes associated to proliferation, activation, inflammation and apoptosis compared to total B cells. We identified and validated 5 receptor/ligand interactions between Bregs and T cells. Functional analysis using specific inhibitors was used to test their suppressive properties and we identified Lymphotoxin alpha (LTA) as a new and potent Breg ligand implicated in Breg suppressive properties.

**Discussion:**

We report for the first time for a role of LTA in GZMB+Bregs as an enhancer of GZMB expression, and involved in the suppressive properties of GZMB+Bregs in human. The exact mechanism of LTA/GZMB function in this specific subset of Bregs remains to be determined.

## 1 Introduction

B lymphocytes are key players in different pathological situations. With their ability to produce antibodies (1), pro-inflammatory cytokines (2) as well as to express MHC and costimulatory molecules (3), they are able to drive and maintain immune response (4). However, over the last decade, there are increasing evidences of the existence of B cells with suppressive properties, also known as regulatory B cells (Bregs) (5). IL-10 producing B cells were the first to be described in mice model of experimental autoimmune encephalomyelitis as a modulator of the pathogenic Th1 responses (6). This Breg subset was later found to be protective against rheumatoid arthritis in mouse (7) and human (8). *in vitro* , IL-10 producing B cells have suppressive functions on different immune cell subsets including T cells, dendritic cells and monocytes, and by promoting Tregs proliferation (9). Granzyme B expressing B cells (GZMB+Bregs) are another population of Bregs, described more recently in human in situations of cancer (10, 11), auto-immune diseases (12), HIV infection (13). We reported that GZMB+Bregs were increased in transplantation in a situation of tolerance in human kidney transplantation (14) and we showed that they also play a role in healthy volunteers (HVs) where they are involved in the homeostasis of the immune response (14, 15). GZMB+Bregs have demonstrated suppressive properties on CD4+CD25- effector T cells, by inhibiting their proliferation and production of cytokines (14, 16). As IL-10 producing B cells, they can produce IL-10 upon stimulation but IL-10 is not involved in their suppressive properties. We reported that this Breg cell subset was dependent on GZMB protein since GZMB inhibitors did partially restore CD4+CD25- T cell proliferation in coculture assays (14). Nevertheless, this recovery was only partial and no specific marker other than GZMB has so far been identified for this subset as for other Bregs (14, 17).

In this paper, we phenotypically and functionally characterized the population of GZMB+Bregs in HVs. We analyzed the transcriptome of GZMB+Bregs at single cell level and found that they exhibit a specific and differential gene expression profile, mainly associated with cell proliferation, apoptotic and metabolism profile, an enrichment in IgG2 and IgG3, a decrease in IgD and IgM, and an impaired antigen presentation in accordance with their differentiated B cells profile. We analyzed the impact of GZMB+Bregs on CD4+CD25- effector T cell were characterized by strong regulation of genes associated with T cells proliferation, activation, IFN pathway, inflammation, and apoptosis. Finally, we identified and evidenced that Lymphotoxin alpha (LTA) is a novel ligand of GZMB+Bregs not only involved in induction of GZMB+Bregs but also in their suppressive properties on T cells with a direct induction of GZMB expression.

## 2 Materials and methods

### 2.1 Samples and cell isolation

Blood was obtained from healthy adult donors (Etablissement Français du sang, Nantes, France). All samples were obtained under informed consent and in accordance with EFS policies. The procedures described in this manuscript were approved by the French « Ministère de l’Enseignement Supérieur et de la Recherche » (MESR) and « Comité de Protection des Personnes » (CPP) (n°DC-2011-1399).

Peripheral Blood Mononuclear Cells (PBMC) were isolated from whole blood using Ficoll gradient centrifugation using standard procedures. Following red blood cell lysis, cells were frozen in Fetal Calf Serum 10% DMSO in 10m/mL cryovials at -80°C using Corning CoolCell for 24h and at -150°C until experiments.

### 2.2 Culture medium preparation

All cultures were made in RPMI 1640 containing 10% Fetal Calf Serum, 2mM L-Glutamine, 100U/mL penicillin/streptomycin.

### 2.3 Induction of GZMB+Bregs

B cells were enriched using Human B Cell Isolation Kit II (Miltenyi Biotec). Separations were performed on an AutoMACS pro Separator following supplier instructions. GZMB+Bregs being a scarce population in HVs with no specific signature, we separated the B cells in two fractions. The first one was left unstimulated in complete medium without stimulation for 72h at 37°C 5%CO_2_, and the second one was used to induce GZMB expression as previously described (18): CpG ODN 2006 [1µg/mL] (Invivogen), soluble rhCD40L [50ng/mL] (R&D systems), rhIL-2 [50IU/mL] (Proleukine – Novartis), rhIL-21 [10ng/mL] (R&D systems), anti-human IgG/A/M F (ab)’2 [5µg/mL] (Jackson ImmunoResearch)) in complete medium in 6-well plates at 10^6^ cells/mL for 72h at 37°C 5%CO_2_. According to this protocol, >95% GZMB+Bregs express GZMB and display suppressive activity (18). In the following experiments, these cells are thus referred as GZMB+Bregs vs their non-Bregs counterpart.

### 2.4 B/T cell coculture experiments

Bregs induction protocol was modified not to exceed 4 days of culture as keeping cells without stimulation for prolonged times is deleterious for their survival. On the second day of the induction of GZMB+Bregs culture, CD4+ CD25- T cells were enriched from overnight resting PBMCs (10m/mL, 4°C, complete RPMI) by two subsequent negative selections using CD4 T cell isolation kit (Miltenyi Biotec) and CD25+ Microbeads II (Miltenyi Biotec). Separations were performed on an AutoMACS pro Separator following supplier instructions. Autologous purified T cells were then activated with anti-CD3/anti-CD28 dynabeads (beads to T cell ratio of 1:1, Invitrogen), and then incubated for 3 days at 37°C 5% CO_2_ with or without non-Bregs or GZMB+Bregs washed from their stimulation cocktail. Cocultures were performed in 96-well plate in 150µL with 5.10^5^ CD4+CD25- T cells and either 10^5^ resting B cells, 10^5^ GZMB+Bregs or control media. The suppressive properties of GZMB+Bregs was assessed by the analysis of the proliferation of CD4+CD25- T cells. T cells alone were used also as control for basal CD4+CD25- T cell transcriptome. For RNAseq studies, we collected 48 wells of T cells alone, 36 wells of T cells/resting B cells and 36 wells of T cells/enriched GZMB+Bregs per donor.

### 2.5 Cell multiplexing and single cell RNA sequencing

scRNAseq using the CITE-seq method (19) was essentially carried out as described in Abidi et al. (20). For each experiment, cells were marked with viability dye (Fixable Viability Dye eFluor 450, 1/1000 in PBS without azide or protein, Invitrogen) for 25 min. Living cells were sorted on an ARIA III (BD Biosciences) and marked with conjugated DNA sequences (HashTag Oligonucleotide, HTO, Chromium Single Cell 3’ Feature Barcode Kit, PN-1000079) specific of the donor and the experimental condition following CITE-seq protocols (19). Cells were then pooled with similar amounts and 20 000 total cells were loaded onto a Chromium controller (10X genomics) (Chromium Next GEM Single Cell 3’ Kit v3.1, ref PN-1000121; Chromium Next GEM Chip G Single Cell Kit, ref PN-1000120). Libraries were then prepared and sequenced on a Nova-Seq 6000 (Illumina) at the GenoBird platform (IRS-UN, CHU Nantes). Raw reads were analyzed using FastQC for quality controls and were then processed using CellRanger pipeline (v3.1.0) with default parameters). Generated FASTQ files were aligned to the reference genome GRCh38.

### 2.6 scRNA-seq analysis

Analysis was performed using R (version 4.0.4) and Rstudio (version 1.3.1056). Data was further analyzed using the Seurat package (21) (v4.0.2) for the demultiplexing and pre-processing steps. Briefly, cells with less than 200 genes and more than 4000 genes or 25% of mitochondrial genes were excluded. SingleR v1.4.1 was used to identify cells and clusters according to phenotypes as described in the Human Primary Cell Atlas for cocultures (22).

Differential gene expression was calculated with Seurat (21) v4.0.2 and MAST (23) v1.16.0. To only retain genes that were robustly expressed, differential gene expression was calculated only for genes expressed in at least in 20% of cells in one of the two groups (GZMB+Breg and non-Breg cells) retaining 5901 genes. The rationale for choosing a log2 foldchange cut-off to define differentially expressed genes (0.58) was to use at least the same or higher log2 fold change than GZMB expression since this is how these cells were defined in the first place.

Gene ontology analysis was performed with clusterProfiler (24). Gene set Enrichment Analysis (GSEA) was performed with all gene sets from the Hallmark, C3, and C5 libraries of the Molecular signature Database from Broad Institute (http://www.broad.mit.edu/gsea/). Receptor ligand analysis was performed with nichenet (25) v1.0.0 using default parameters with B cells as sender cells and T cells as receiver cells. The data discussed in this publication have been deposited in NCBI’s Gene Expression Omnibus (26) and are accessible through GEO Series accession number GSE224461 as of the time of publication.

### 2.7 Gene expression validation

B cells were purified using StraightFrom® Whole Blood CD19 MicroBeads and cultured for 3 days using an activation cocktail (CpG ODN 2006 [2.5µg/mL], CD40L [50ng/mL], rhIL-2 [50IU/mL], anti-human IgG/A/M F (ab)’2 [2µg/mL]) or a GZMB differentiation cocktail (16) (CpG ODN 2006 [1µg/mL], CD40L [50ng/mL], rhIL-2 [50IU/mL], rhIL-21 [10ng/mL], anti-human IgG/A/M F (ab)’2 [5µg/mL]). Cell pellets were resuspended in RLT buffer (Qiagen) containing 1% β-mercaptoethanol before subsequent RNA extraction using a RNeasy Micro Kit according to the manufacturer’s instructions (Qiagen). RNA quality and quantity were assessed by spectrometry (Nanodrop) and electrophoresis (Agilent RNA 6000 Pico Kit). Transcriptomic profiling was performed using nCounter-Human-Immunology-V2 Panel and nCounter SPRINT Profiler. Gene expression data analysis was performed using the nSolver Analysis Software (v4.0). Only reference genes with expression levels less than 2-fold in as compared to pre-culture settings were used for the normalization (*ABCF1, EEF1G, POLR2A, PPIA, RPL19, SDHA, TBP*).

### 2.8 Western blots

Validation of gene expression in GZMB+Bregs was performed by western blotting from GZMB+Bregs or non-Bregs. After GZMB+Bregs induction cultures, cells were washed 3 times in PBS and lysed in 1X RIPA solution with protease inhibitors (Thermofisher scientific) on ice for 30 minutes followed by a thermal shock. 10µg total protein samples in 15µ LPBS + 5µL Laemmli buffer 4X (Bio-Rad, Cat#1610747) were heated for 5 minutes at 95°C and then separated by SDS-PAGE with 2.5µL DTT (Bio-Rad, Cat #16106747) 4-20% gels (mini-PROTEAN® TXG™ Precast Protein Gels, Bio-Rad, Cat #4561093). Total proteins were transferred to a nylon membrane using the TransBlot Turbo Transfer System (Bio-Rad, Cat #1704150). Membrane was saturated with a solution of Tris-buffered saline 0.1% Tween 5% milk at 4°C overnight. immunoblotting was then performed for MIF (1:500) (RRID : AB_2788655, Cat #PA5-81446; Invitrogen), Galectin-3 (1:1000) (RRID : AB_837131, Cat #14-5301-81; Invitrogen), lymphotoxin-α (1:500) (RRID : AB_2900689, Cat #PA5-116055; Invitrogen) and GAPDH (1:10000) (RRID : AB_1078991, Cat #G8795, Sigma-Aldrich) was performed (2h, room temperature). After 3 washes in TBS-Tween 0.1%, membranes were incubated with secondary antibodies for 1h at room temperature. Revelation was performed with the superSignal West Atto kit (ThermoFisher Scientific, Cat #A38554) on a Bio-Rad ChemiDoc MP Imaging System (Bio-Rad, Cat #17001402).

### 2.9 LTA and GAL3 inhibition assays

To evaluate LTA and GAL3 pathways in GZMB+Bregs function, we used the coculture model with CPD-labeled CD4+CD25- effector T cells (CPD eF450, ThermoFisher scientific). T cell proliferation was evaluated as the percentage of CPD^low^ T cells on day 3 by flow cytometry. To study the roles of different signaling pathways in the suppressive effect of B cells, different inhibitors, including GZMB inhibitor (GZMB inhibitor IV, Calbiochem Research Biochemicals), Galectin-3 inhibitor (MCP, EcoNugenics, kindly donated by Louis Boutin (UMR 942)), and Lymphotoxin-α inhibitor (Pateclizumab, Absolute Antibody), were added either during the B cell pre-stimulation, during the coculture or in both steps. When not specified, all inhibitors were used at 10µg/mL. The activity of the inhibitors was also assessed on GZMB expression by B cells expression of GZMB (anti-GZMB PECy7-conjugated antibody, Biolegend) and the proliferation of GZMB+Bcells (CPD eF450, ThermoFisher scientific) in the 3-days culture model.

### 2.10 Statistical analysis

Differentially Expressed Genes (DEG) p values were calculated by the using seurat FindMarkers function using a hurdle model tailored to scRNAseq data from MAST R package on R. Cytometry data were analyzed with flowJo v10. Western blot data were quantified using ImageJ (27). One-way ANOVA with Tukey’s test for multiple comparisons and Khi^2^ were performed with GraphPad Prism 9. Differences were defined as statistically significant when P<0.05 (*), P<0.01 (**), P<0.001 (***) and P<0.0001 (****). P values were adjusted to the number of tests using FDR adjustment. Data are presented as mean values ± standard error of the mean (SEM).

## 3 Results

### 3.1 GZMB+Bregs are skewed toward a differentiated plasmablasts/plasma cell phenotype

**Figure 1:**
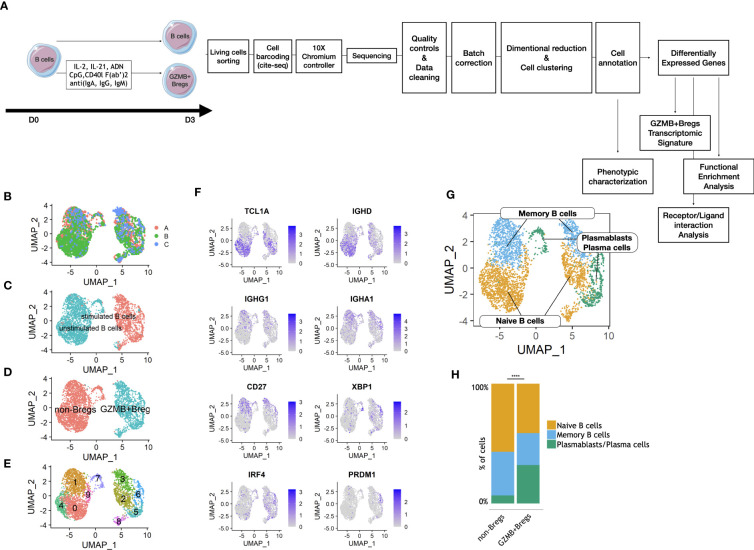
Clustering and phenotyping of GZMB+Bregs. Schematic view of the study design from the cell preparation for scRNAseq. GZMB+Bregs or non-Bregs were induced from B cells isolated from PBMC. Living cells were then marked with anti-β2-microglobulin or anti-CD293 conjugated DNA sequences specific of the donor and the experimental condition following CITE-seq protocols. Libraries were then prepared and sequenced on a Nova-Seq 6000 (Illumina) in 3 different runs: 1,989 cells in run 1, 3,919 cells in run 2 and 1,493 cells in run 3. Data were corrected for batch effect and analyzed on R using the Seurat library. After dimensional reduction and cell clustering, cells were annotated based on their expression of phenotypic markers to characterize B cell subpopulations in GZMB+Bregs as compared to non-Bregs. Th differentially expressed genes were calculated on the 5901 genes expressed at least in 20% of cells in one of the two compared groups. Only genes with a Log2(Fold-change) > 0.58 (corresponding to the Fond-Change of GZMB) and an adjusted p.value <0.05 were selected for the transcriptomic signature of GZMB+Bregs and functional enrichment analysis **(A)**. Batch effect was corrected by combining cells from the 3 different runs with reciprocal PCA. Transcriptomes of Bregs and non-Bregs cells were analyzed using an unsupervised dimensionality reduction algorithm (UMAP) to identify groups of cells with similar gene expression profiles. Each point represents a cell and is represented in color by its Batch **(B)**. **(C–E)** Transcriptomes of Bregs and non-Bregs cells from 4 HVs were analyzed using an unsupervised dimensionality reduction algorithm (Seurat) to identify groups of cells with similar gene expression profiles. Each point represents a cell and is represented in color by its stimulated or unstimulated status as characterized by HTO **(C)**, unsupervised **(D)** or supervised **(E)** clustering. **(F)** Expression of markers used for phenotypic classification of naive and immature B cells (TCL1A, IGHD), memory B cells (IGHG1, IGHA1, CD27), and plasmablasts/plasma cells (XBP1, IRF4, PRDM1, CD27). **(G)** Phenotype of the naive, memory, and plasmablasts/plasma subset of B cells as represented in UMAP. **(H)** Percentage of naive, memory, and plasmablasts/plasma cells in non-Bregs and GZMB+Bregs metaclusters were analyzed using Khi2-test. The plasmablasts were significantly enriched in GZMB+Bregs (p<0.0001). Differences were defined as statistically significant with p<0.0001 (****).

We first characterized the transcriptomic phenotype of GZMB+Bregs at the single cell level (**Figure 1A**). Sorted total B cells were activated with a cocktail specifically triggering B cells toward a GZMB+Breg phenotype (IL-2, IL-21, anti-human IgG+A+M, CpG, CD40L) as previously described (18) or left unstimulated. After 3 days, scRNAseq was performed on living cells to characterize the transcriptome of enriched GZMB+Bregs (>95% GZMB+Bregs) compared to unstimulated B cells containing more than 99% non-Bregs (16) (see methods). Experiments were conducted using blood from 4 HVs in three different experiments. After QC and doublet removal, a total of 3,570 cells were used for the analysis. The three datasets were combined using reciprocal PCA to reduce batch effect, read counts were normalized and scaled. After clustering and dimensional reduction using UMAP, batch integration was successful as all three runs were well integrated, i.e., neither of the three batches formed isolated clusters (**Figure 1B**). Interestingly, 99% of stimulated cells clustered together, with less than 5% of unstimulated cells (**Figure 1C**) indicating that they display an inherent distinct transcriptomic profile. B cells were manually annotated, based on the proportions of unstimulated total B cells and enriched GZMB+Bregs in each cluster (**Figure 1D**). To further explore whether there are B cell subtypes present in the two large clusters, additional clustering was performed resulting in 5 non-Bregs clusters (0, 1, 4, 7, 9) and 5 GZMB+Bregs clusters (2, 3, 5, 6, 8) (**Figure 1E**). Based on marker gene expression, three subpopulations were determined in non-Bregs: naïve B cells expressing *TCL1A* and *IGHD*, coding for the heavy chains for IgD, but not *CD27;* memory B cells expressing *CD27* and the heavy chains for IgG and IgA (*IGHG1* and *IGHA1*, respectively) and a small cluster of plasmablasts/plasma cells expressing *CD27, XBP1*, and *IRF4* (**Figure 1F**). The same three subpopulations were identified in GZMB+Bregs, indicating that the three B cell subtypes are present in both, GZMB+Breg and non-Bregs (**Figure 1G**). We next quantified the relative proportions of the three subtypes in both conditions and revealed that GZMB+ Bregs were highly enriched (Fold change= 4.7, Statistical test Khi^2^, p-value < 0.0001) in plasmablasts/plasma cells, suggesting that GZMB+Bregs are skewed towards a differentiated B cell profile (**Figure 1H**).

### 3.2 GZMB+Bregs harbor a specific transcriptional profile involved in proliferation and metabolism, decreased B cell activation as well as antigen presentation

**Figure 2:**
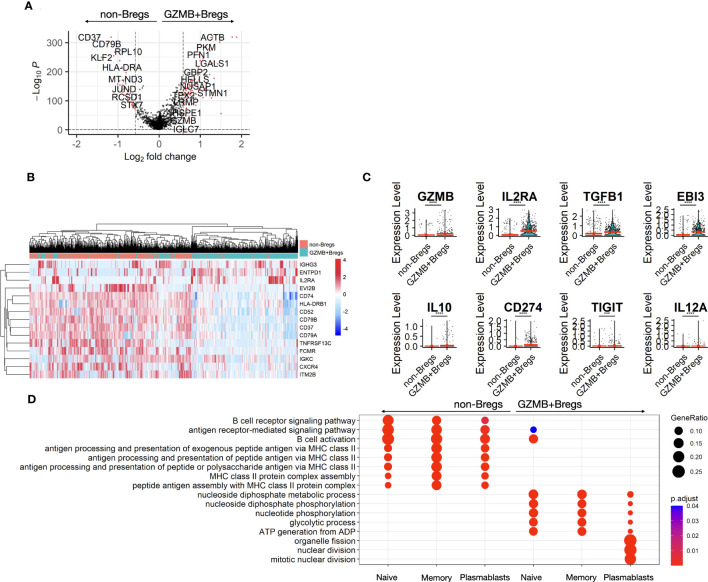
B cell differential expression. The two clusters of GZMB+Bregs and non-Bregs were analyzed using differential expression methods (Seurat, MAST) to identify DEGs represented in volcano plot. Each point represents a gene by its fold change difference between the two clusters and the adjusted p.value **(A)**. Among the genes with a fold change greater than GZMB, the 15 associated to cell-surface proteins were represented as a heatmap **(B)**. Different Bregs subsets have been characterized by several markers with variable expression in this dataset **(C)**. Gene ontology analysis was performed on the 104 up- and 45 down-regulated DEGs. Redundent terms were then reduced using the rrvgo package (1.8.0). **(D)** Differential gene expression and their ontologies were also analyzed within the naive, memory and plasmablasts/plasma cells and represented in dotplots. Differences were defined as statistically significant when P<0.001 (***) and P<0.0001 (****).

To identify what distinguishes the GZMB+Bregs from non-Breg cells a differential gene expression analysis was performed to identify gene signatures associated with GZMB+Bregs (see Methods). 149 genes were differentially expressed between GZMB+Bregs and non-Bregs cells with 104 genes up and 45 down in GZMB+Bregs (FDR Q-value < 0.05, **Figure 2A**). Among the most downregulated genes, *CD37*, *CD79B*, and *CD74* are surface receptors. PNRC1 and KLF2 are transcription modulators, all associated to lymphoid cell activation whereas the upregulated genes in GZMB+Bregs (*KI67*, *CCND2*, *HMGB2*, and *LGALS1*) are associated with cell proliferation and chemotaxis. Next, we investigated specifically the differential expression of cell surface markers. Notably, we found 3 (*ENTPD1*, *IL2RA*, and *IGHG3*) and 14 (*EVI2B*, *CD37*, *CD52*, *CD74*, *CD79A*, CD79B, *TNFRSF13C*, *HLA*-*DRB1*, *HLA*-*DPB1*, *HLA*-*DRA*, *IGKC*, *CXCR4*, *FCMR*, and *ITM2B*) surface markers genes that were respectively up- and down-regulated in GZMB+Bregs vs non-Breg cells, indicating that the phenotypic display of surface markers is drastically altered in GZMB+Bregs (**Figure 2B**). We note that B cell markers reported by us and other groups previously described to be associated with different subpopulations of Bregs (9, 28, 29), such as *GZMB*, *IL2RA* coding for CD25, *TGFB1*, and *EBI3* (coding for a chain of IL-35) were found significantly overexpressed in GZMB+Breg clusters, showing consistency between prior results and single cell transcriptome (**Figure 2C**). We note that other previously reported genes such as IL-10, *CD274*, *TIGIT*, and *IL12A* (the other chain of IL-35) were expressed in less than 5% of GZMB+Bregs (**Figure 2D**), which may be due to technical limitations of scRNA-seq or differences between mRNA and protein expression.

In order to better characterize GZMB+Bregs gene expression, pathway enrichment analysis was performed on the 149 genes specific of GZMB+Bregs focusing on known pathways and functions using gene ontology analysis. The gene ontologies associated with the 104 up-and and 45 downregulated genes of the GZMB+Bregs signature were reduced in a list of non-redundant terms. Among the most enriched and recurrent terms, GZMB+Breg signature was found to be enriched for cell cycle (*NUSAP1*, *SMC2*, *MKI67*, *PTTG1*, *SMC4*, *RAN*, *UBE2C*, *TPX2*, *CENPF*, *RANBP1*, *UBE2S*), metabolism (*GAPDH*, *TPI1*, *ENO1*, *LDHA*, *DDIT4*, *PGK1*, *ALDOC*), cellular response to interferon-gamma (*ACTG1*, *GAPDH*, *GBP2*, *VIM*, *STAT1*, *HSP90AB1*) (**Figure S1A**) whereas downregulated genes were associated with B cell activation (*CD74*, *CD79A/B*, *PRKCB*, *TNFRSF13C*, *ZFP36L2*, *IGKC*, *BANK1*), and MHC class II antigen presentation (*CD74*, *HLA-DRA*, *HLA-DRB1*, *HLA-DPB1*, *HLA-DMB*) (**Figure S1B**). To further tease out specific gene expression profiles associated to the three sub-populations, differential gene expression between GZMB+Bregs and non-Breg were performed on the naive, memory, and plasmablasts/plasma cell subclusters separately. There was a downregulation of genes associated with antigen presentation, B cell activation, and metabolism in all three GZMB+Bregs subtypes. Interestingly, only the genes associated with cell cycle (i.e., nuclear division) were found specifically enriched in the plasmablasts/plasma cells, in accordance with the high proliferative ability of plasmablasts (**Figure 2D**).

To confirm gene ontology results, transcriptomic signatures of major functions in B cells were assessed using GSEA. GZMB+Bregs were characterized by the downregulation of genes associated with MHC class II antigen presentation (adjusted p-value < 0.001, Normalized enrichment score (NES) = -2.13; **Figure S2A**), upregulation of genes associated with apoptotic signaling pathway (p <0.01, NES = 1.44; **Figure S2B**), and cell proliferation (p < 0.001, NES = 1.50; **Figure S2C**) compared to non-Bregs. While we found no significant association for genes associated with immunoglobulin production (p=0.06, NES = -1.3; **Figure S2D**), and no significant differential expression for *IGHG1*, *IGHG4*, *IGHA1*, and *IGHA2* expression between the two B cell clusters (**Figure S2E**), we found an enrichment in genes coding for IgG2 and IgG3 in naïve, memory, and plasmablasts GZMB+Bregs and a decrease in genes coding IgD and IgM in naïve GZMB+Bregs (p<0.0001 vs non Bregs), still in accordance with their enrichment toward plasmablasts/plasma cell profile.

Altogether, we found that regulatory B cells displayed a specific gene expression profile, mainly associated with cell proliferation, apoptosis, and an altered metabolism. In addition, there was a marked enrichment in IgG2 and IgG3 and a decrease in IgD and IgM, with impaired antigen presentation in accordance with their matured differentiation status.

### 3.3 GZMB+Bregs mediate their regulatory function by downregulating CD4+CD25- T cells genes implicated in T cell activation, IFN pathway and inflammation during *in-vitro* coculture

**Figure 3:**
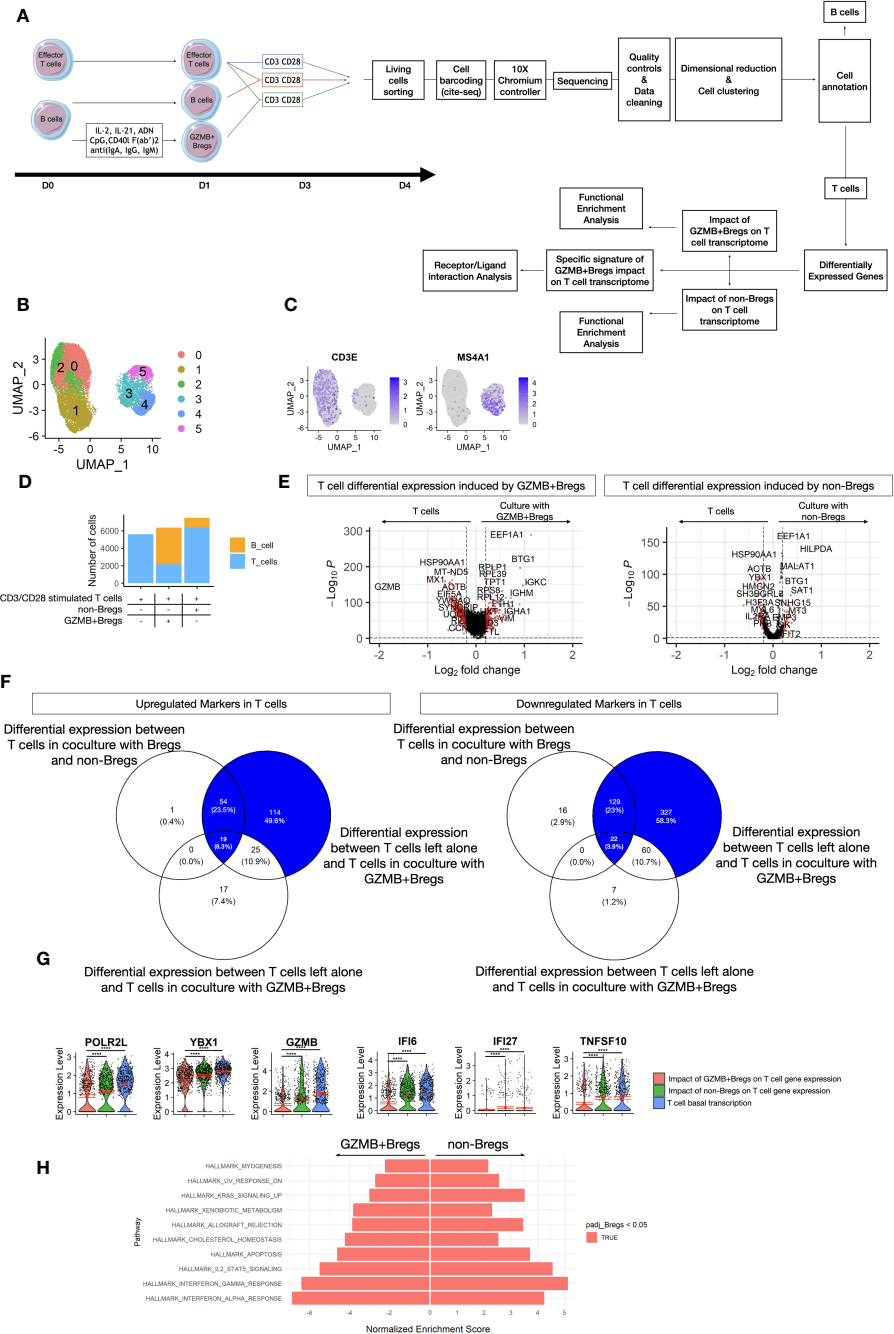
T cells coculture dataset. **(A)** Schematic view of the study design from the cell preparation for scRNAseq. GZMB+Bregs or non-Bregs were induced from B cells isolated from PBMC for 1 day. CD3/CD28 activated T cells were cultivated alone, with non-Bregs or GZMB+Bregs for 3 days. At the end of the cultures, living cells were marked with anti-β2-microglobulin or anti-CD293 conjugated DNA sequences specific of the donor and the experimental condition following CITE-seq protocols. Libraries were then prepared and sequenced on a Nova-Seq 6000 (Illumina). After dimensional reduction and cell clustering, cells were annotated based on their expression of phenotypic markers to characterize B cell and T cells. After 3 days of culture in absence of Bregs induction cocktail, the expression of GZMB was not differential between GZMB+Bregs and non-Bregs. Thus, the B cells were not analyzed. Differentially expressed genes were calculated on the 5901 genes expressed at least in 20% of cells in one of the two compared groups. Only genes with a Log2(Fold-change) > 0.2 and an adjusted p.value <0.05 were selected for the transcriptomic signature of GZMB+Bregs and functional enrichment analysis. The signature of GZMB+Bregs impact on T cell transcriptome was described as the differentially expressed genes between T cells in culture with or without GZMB+Bregs and not differentially expressed in culture with or without non-Bregs, unless the genes were also differentially expressed in T cells in culture with GZMB+Bregs or non-Bregs. **(B–H)** Transcriptomes of Bregs and non-Bregs cells from 2 HVs were analyzed using an unsupervised dimensionality reduction algorithm (Seurat) to identify groups of cells with similar gene expression profiles. Each point represents a cell and is represented in color by its unsupervised clustering **(B)** and Expression of phenotypic markers CD3E and CD20 **(C)**. The proportions of B and T cells within each condition was represented as a Histogram of the number of B cells and T cells within each condition **(D)**. The imprints of GZMB+Bregs and non-Bregs on T cell transcriptome were analyzed using differential expression methods (Seurat, MAST) to identify DEGs represented in volcano plot. Each point represents a gene by its fold change difference between the two clusters and the adjusted p.value **(E)**. The specific imprint of GZMB+Bregs was determined using Venn diagram to exclude the 85 genes also modulated by non-Bregs with no exacerbated expression between the two conditions of T cells in culture with GZMB+Bregs or non-Bregs **(F)**. The 6 most downregulated genes are represented in violin plots **(G)**. Differences were defined as statistically significant when P<0.0001 (****). **(H)** GZMB+Bregs and non-Bregs induce opposite effect on effector-T cell related pathways and non-immune related cell-activation pathways from the Hallmark library as represented by their Normalized Enrichment Score from GSEA.

To characterize the imprint of GZMB+Bregs on effector T cells, purified CD4+CD25- T cells from HVs were activated with anti-CD3/CD28 in the presence of GZMB+Bregs, non-Bregs at a ratio 1:5 B vs T cells or left alone for 3 days (see methods, **Figure 3A**). Single cell transcriptomics was then carried out on all living cells in the three culture conditions. In total 19,212 cells (8,131 cells from HV1 and 11,081 cells from HV2) were analyzed after quality controls and exclusion criteria (see methods). Clustering and dimensional reduction using UMAP revealed two major clusters which could be further divided into 5 clusters (**Figure 3B**). Manual annotation was then carried out, according to their expression of T and B cells phenotypic markers (CD3E, MS4A1) and which show that clusters 0, 1, 2 were T cells and clusters 3 to 5 were B cells (**Figure 3C**). Quantifications of absolute cell numbers per condition, revealed that lower numbers of CD4+CD25- T cells were found when cocultured with GZMB+Bregs (**Figure 3D**), which is in accordance with their suppressive properties and previous reports. Next, we carried out differential gene expression analysis of the T cells. 750 genes were differentially expressed in CD4+CD25- T cells cocultured with GZMB+Bregs vs CD4+CD25- T cells alone (**Figure 3E**, log2Fc =0.2, FDR Q-value <= 0.05). 85 genes (25 up- and 60 down-regulated, respectively) were excluded because they were also differentially expressed when CD4+CD25- T cells were cocultured with non-Bregs (**Figure 3F**). 665 genes were thus specifically associated with CD4+CD25- T cells in coculture with GZMB+Bregs, with significant downregulation of the activation genes *POLR2L*, *YBX1* and inflammatory genes such as *GZMB*, *IFI6*, *IFI27*, *TNFSF10* (**Figure 3G**).

GSEA analysis was performed to assess the impact of GZMB+Bregs and non-Bregs on T cell pathways. Both GZMB+Bregs and non-Bregs cocultures were associated with a decrease of gene pathways of T cell proliferation (E2F targets, MYC targets, G2M checkpoint, and mitotic spindle), activation (PI3K-AKT-MTOR MTORC1, WNT, IL-6), and metabolic pathways (Fatty acid metabolism, Oxidative phosphorylation) (**Figure S3**). In contrast, the immune-related gene sets IL-2-STAT5, Interferon-α, -γ, and KRAS signaling pathways, T cell apoptosis, and a set of genes increased during allograft rejection were found down-regulated only when CD4+CD25-T cells were cocultured with GZMB+Bregs (**Figure 3H**). Altogether these data show that effector T cells cocultured with GZMB+Bregs are characterized by a down regulation of genes associated with T cells proliferation, activation, IFN pathway, inflammation, and apoptosis.

### 3.4 Identification of receptors-ligands involved in CD4+CD25- T cells and GZMB+Bregs interactions

**Figure 4:**
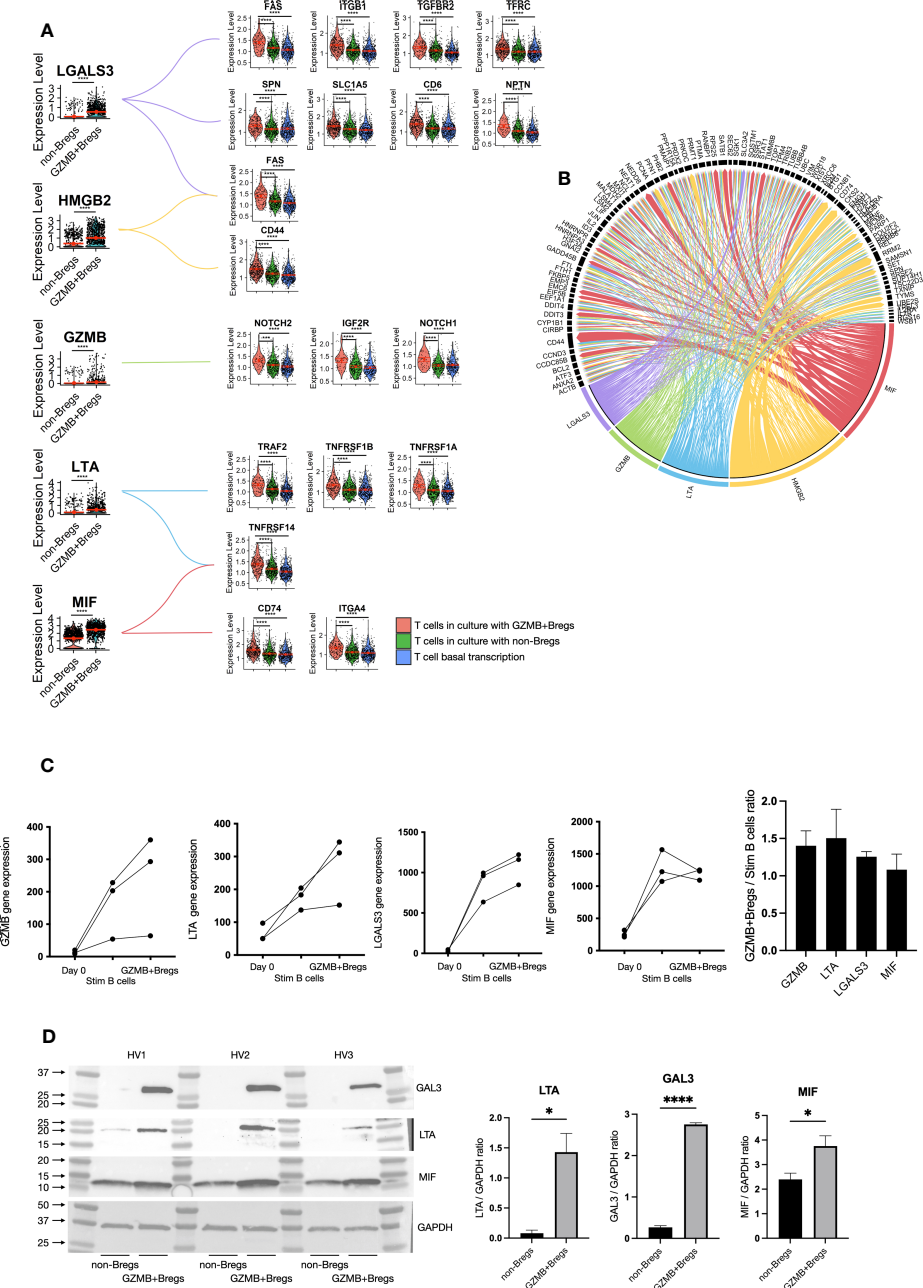
GZMB+Bregs interactions with T cells. To understand regulatory B cells mechanisms, we used the nichenet algorithm that predict the interaction of B cell ligands with receptors on the T cell based on T cell DEGs and prior knowledge on the transcriptomic changes downstream receptor-ligand interaction. Among the 149 DEGs in GZMB+Bregs compared to non-Bregs, LGALS3, GZMB, LTA, HMGB2 and MIF were predicted to be potent inducers of T cell differential expression. **(A)** B cell expression of predicted ligands and their receptors are represented in violin plots. **(B)** Predicted ligand induced gene transcription is represented as a chord diagram. Arrow widths represents the strenght of the interactions. **(C, D)** LTA, LGALS3 and MIF gene expression were validated using the nCounter® SPRINT Profiler in GZMB+Bregs compared to activated B cells and B cells before the stimulation with the GZMB as a control **(C)** and at the protein level by western blot **(D)**. RNA and protein analysis were independently performed on B cells from 3 HVs. Data represent each independent experiment. Data represent mean ± SEM. Differences were defined as statistically significant by t.test when P<0.05 (*) and P<0.0001 (****).

To further characterize how GZMB+Bregs mediate their regulatory effects on CD4+CD25- effector T cells, we analyzed receptor-ligand interactions using Nichenet (25), which enables to link differentially expressed genes in target cells (i.e. receptors and their known downstream signaling genes) to differentially expressed ligands from source cells using known ligand-receptor interaction data sources. For this, we defined the T cells as target expressing cells and B cells as ligand expressing cells, and used the 149 differentially expressed GZMB+Bregs genes as potential ligands (see **Figure 2**) and the 665 differential genes specific to CD4+CD25- T cells cocultured with GZMB+Bregs as targets genes (see **Figure 3**). Five differentially expressed ligands were found in GZMB+Bregs to interact with receptors expressed on T cells: Granzyme B (GZMB) interacts with NOTCH1, NOTCH2, and IGF2R. Galectin-3 (*LGALS3* (gene), GAL3 (protein)) interacts with FAS, ITGB1, TGFBR2, TFRC, SPN, SLC1A5, CD6, and NPTN. Macrophage migration Inhibitory Factor (MIF) interacts with CD74, NPTN an TNFRSF14. High Mobility Group Box 2 (HMGB2) interacts with CD44 and FAS. Lymphotoxin-α (LTA) interacts with TNFRs (TNFRSF1A, TNFRSF1B, TNFRSF14, and TRAF2). This was concomitant with an upregulation of their corresponding receptors in CD4+CD25- T cells when co-cultured with GZMB+Bregs (**Figure 4A**). Nichenet identified additional target genes downstream of the receptors in the CD4+CD25- T cells indicating that the signaling pathways downstream of the ligand receptor interaction are also altered (**Figure 4B**). It is noteworthy that the target genes of the different ligands were redundant, indicating that the five ligands may not be implicated in the B/T interactions.

Interestingly, GZMB was one of the five ligands which was found in the GZMB+Bregs. GZMB is expressed in the majority of GZMB+Bregs (95%) independently of their annotation (naïve, memory or plasmablasts/plasma cells) and the role of GZMB was already demonstrated notably at the protein level. Thus, this result acts as proof of concept highlighting that this approach enables the detection of receptor-ligand pairs without prior knowledge. The second ligand, HMGB2 was found to only be upregulated in plasmablasts/plasma cells (**Figure S4**) and there is currently no specific inhibitor available for this protein, thus HMGB2 was not further investigated in this study. Ligand expression was then measured in GZMB+Bregs with regulatory properties and as control, in unstimulated B cells and B cells stimulated without IL-21 that are unable to inhibit CD3/CD28 activated T cells proliferation. We next aimed to validate the differential gene expression of *LGALS3*, *LTA*, and *MIF* in independent experiments using q-PCR on B cells isolated from three distinct HV (**Figure 4C**). We found that indeed, *LGALS3*, *MIF*, and *LTA* gene expression was upregulated in GZMB+Bregs as compared to freshly isolated B cells (day 0). As this upregulation could merely be due to B cell activation, we also examined *LGALS3*, *MIF*, and *LTA* expression in activated B cells with goat anti-human IgG/IgA/IgM (H+L) F (ab)’2, IL-2, CD40L, and CpG DNA. These cells are GZMB^low^B cells with no regulatory activity while they are proliferating (CPD^low^) and expressing high levels of activation markers (CD25) (*data not shown*). Expression was not significative different between GZMB^low^B cells and GZMB+Bregs, but both *LGALS3* and *LTA* expression was further increased in GZMB+Bregs in the three HV. This indicates that stimulation and regulatory function have an additive effect on the transcriptomics levels of these receptors. Next, we analyzed protein levels of these three ligands within the two B cell populations (GZMB+Bregs and non-Bregs) in three other HVs. While GAL3 (Fold change = 10.3, paired t-test p value < 0.0001) and LTA (Fold change = 17.7, paired t-test p value = 0.0128) were nearly exclusively expressed in GZMB+Bregs, MIF (Fold change = 1.56, paired t-test p value = 0.0497) was also detectable in non-Breg cells making it a poor candidate for GZMB+Breg-specific regulatory function (**Figure 4D**). In summary, receptor-ligand interaction analysis and subsequent validation experiments identified two new ligands which are promising candidates of regulatory activity in GZMB+Bregs.

### 3.5 GZMB+Bregs function is dependent on LTA

**Figure 5:**
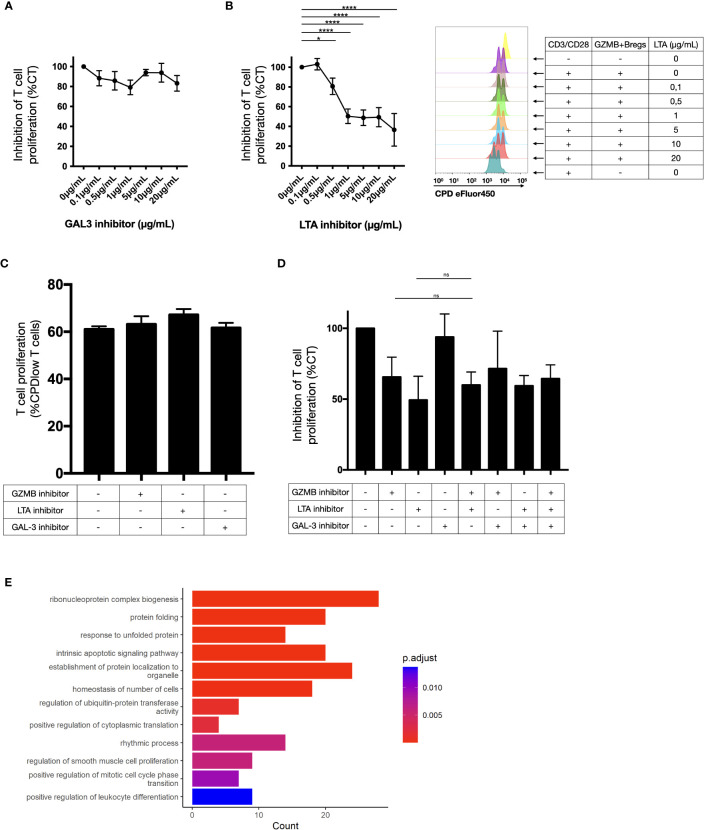
Ligand activity on CD4+CD25- T cell proliferation. **(A–D)** T cell proliferation was measured by flow cytometry (as the percentage of CPD-low T cells) after 3 days-CD3/CD28 stimulation in presence of regulatory B cells in response of increasing doses of galectin 3 inhibitors in coculture assay **(A)** and LTA inhibitors in coculture assay with cytometry proliferation data for one representative donor **(B)**, on T cells alone **(C)** and with different combinations of these inhibitors **(D)** in 6 healthy donors. The values were normalized to the inhibition induced by GZMB+Bregs without any inhibitor. On T cells alone, the proportion of cells that did one or more divisions was around 100% for each condition. That does not permit to visualize a potential proliferation induced by the inhibitors. Thus, we gated on cells that did two divisions or more. No significant difference was found between the CD3/CD28 stimulated T cells alone or with any of the inhibitors. Data represent mean ± SEM. ANOVA was performed with Tukey’s multiple comparison test. Differences were defined as statistically significant when P<0.05 (*) and P<0.0001 (****). **(E)** Gene ontology analysis was performed on the 45 genes downregulated in T cells and linked to LTA activity in the receptor-ligand analysis. Bar plots represent the number of gene and the adjusted pvalue associated to the ontologies.

In a previous study we showed that GZMB blockade only partially recovered the suppressive properties of GZMB+Bregs (14). We thus investigated whether GAL3 and LTA were also relevant for the suppressive properties of GZMB+Bregs. Different combinations and doses of corresponding inhibitors (Pateclizumab for LTA, Modified Citrus Pectin (MCP) for GAL3) were tested in GZMB+Breg/CD4+CD25- T cell cocultures. Whereas GAL3 inhibitor MCP had no effect (**Figure 5A**), LTA inhibitor Pateclizumab significantly reduced the inhibition mediated by GZMB+Bregs in a dose dependent manner (**Figure 5B**). We note that addition of increasing doses of Pateclizumab was not associated with increased B cell mortality (data not shown) and Pateclizumab had no effect on CD3/CD28 activated CD4+CD25-T cells alone, showing that the effect of LTA on T cells was mediated via GZMB+Bregs (**Figure 5C**). Finally, no additive effect was found with LTA and GZMB inhibitors at all the doses tested (**Figure 5D**).

Since the LTA inhibitor blocked the regulatory activity of GZMB+Bregs, we investigated the transcriptomic impact of LTA on T cells. For this purpose, we investigated the function of the known LTA targets (45 genes, see **Figure 4B**) which were identified in the receptor-ligand analysis. Gene ontology analysis of these 45 LTA targets genes indicates that they are implicated in proliferation (mononuclear cell proliferation, cell cycle, cell division, and chromosome organization), apoptosis and cell activation (**Figure 5E**). This indicates that LTA likely mediates its regulatory effects on T cells via the receptors expressed on T cells (TNFRS14, TNFRS1 and TNFRS1B, see **Figure 4A**) and their downstream signaling cascade implicated in proliferation and cell cycle. In summary, we characterized LTA as a novel ligand implicated in the regulatory function of GZMB+Bregs cells.

### 3.6 LTA modulates GZMB expression on B cells

**Figure 6:**
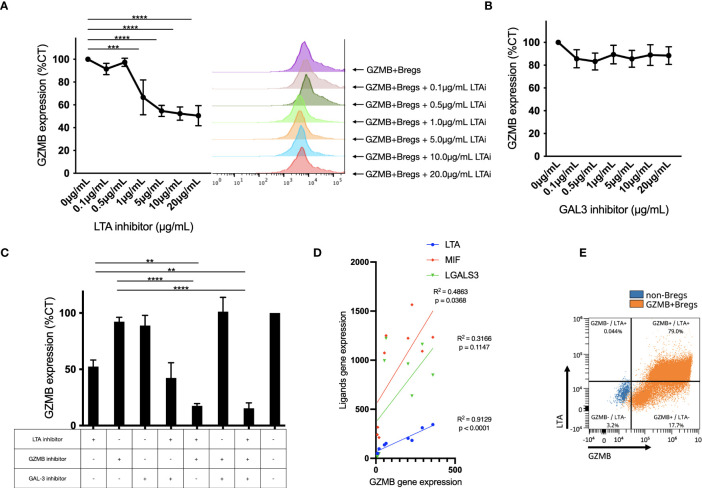
Ligand activity on regulatory B cells. **(A-C)** The expression of GZMB in regulatory B cells in response of increasing doses of LTA with cytometry data from one representative donor **(A)** and galectin 3 **(B)** inhibitors, and different combinations of these inhibitors **(C)** was assessed by flow cytometry in B cells after the 3-days Bregs induction for 6 healthy donors. For each donor, the mean fluorescent intensity was normalized to the condition without any inhibitor. **(D)** The correlation between GZMB mRNA expression and ligands mRNA expression (LTA, LGALS3 and MIF) was assessed using the nCounter® SPRINT Profiler in different B cell subsets (B cells enriched from blood, activated B cells in non-regulatory phenotype and GZMB+Bregs) in 3 different donors. LTA expression is highly correlated to GZMB (R2 = 0.9129) whereas MIF and LGALS3 were not/weakly correlated to GZMB (R2 = 0.4863 and 0.3166 respectively). **(E)** A representative FACS plot of 3 experiments for GZMB and LTA coexpression. Data represent mean ± SEM. ANOVA was performed with Tukey’s multiple comparison test. Differences were defined as statistically significant when P<0.01 (**), P<0.001 (***) and P<0.0001 (****). LTAi, LTA inhibitor Pateclizumab.

To further characterize the role of LTA in GZMB+Bregs we investigated how its gene expression is regulated. Transcription factors (TF) are key regulators of cell fate and differentiation and as of now, none has been associated in the literature with a specific regulatory B cell lineage. To explore this, we used the geneHancer database (30) and the TFBSTools R package (31) to recover transcription factors known to bind to LTA and GZMB promoters. Among the 278 and 132 TFs associated to the promoter regions of LTA and GZMB respectively, two TFs were also upregulated in the GZMB+Bregs (*BATF*, *CREM*) (**Figure S5**). BATF has been shown to act downstream of IL-21R to induce GZMB expression (32). CREM expression was shown to be dependent on CREB phosphorylation in response to receptor activation including TNFR1, one of the receptors for LTA (33). It is thus possible that LTA may be regulating the expression of CREM via autocrine activation of TNFR and thus control its own gene as well as the GZMB gene. To test this, we analyzed the effect of Pateclizumab on GZMB protein expression in GZMB+ Bregs when cocultured with CD4+CD25- T cells for 3 days (**Figure 6A**). Interestingly, LTA inhibition in GZMB+Bregs not only decreased induction of GZMB+Bregs (60% decrease of GZMB+Bregs at 3 days) but also caused a concentration dependent and significant decrease of GZMB expression. It is noteworthy that MPC, the GAL3 inhibitor, had no effect on GZMB protein levels (**Figure 6B**). GZMB and LTA inhibitors had an additive effect on GZMB inhibition in GZMB+Bregs (**Figure 6C**). This dependence of GZMB with regards to LTA was further supported by the strong correlation between LTA and *GZMB* gene expression (Pearson R^2 = ^0.91) as measured using the nCounter® SPRINT Profiler (**Figures 6D, E**). Taken together, these data indicate that LTA regulates the induction of GZMB in GZMB+Bregs and the expression of GZMB in GZMB+Bregs, possibly via an autocrine feedback loop mediated through the TF CREM.

## 4 Discussion

We previously reported that B cells expressing GZMB inhibit the proliferation of effector CD4+CD25- T lymphocytes in a contact and GZMB-dependent manner (16).

Despite several attempts to identify markers specific for regulatory B cells including GZMB+Bregs, no specific nor common markers have been discovered yet (17, 34–36), in accordance with their extensive heterogeneity and the absence of identification of specific lineage for this regulatory lymphocyte subset (9). Finally, GZMB+Bregs only represents about 1-2% of circulating B cells in HVs, which makes identification and isolation of such cells very challenging. We and others found methods to enable the induction of such Bregs cells while keeping their phenotype and suppressive properties thus offering the possibility to obtain highly purified Breg cells (more than 95% of highly purified GZMB^+^Breg cells in our hands) and opening the way to potential future clinical applications (18, 37–39). We took advantage of this protocol to better characterize this GZMB+Breg population phenotypically and functionally. In this purpose, we analyzed the transcriptome of GZMB+Bregs in HVs at the single cell level, we analyzed their impact on CD4+CD25- effector T cell transcriptome and identified potential T/GZMB+Bregs receptor-ligand interactions with a putative role in their suppressive properties.

scRNAseq was preferred because it is particularly appropriate to study the heterogeneity of GZMB+Breg cell populations. Our results indicate that GZMB+Bregs cells are found in all B cell subsets at different stages of B cell differentiation (naïve, memory and plasma cells) with a significant enrichment in more differentiated B cells. Yet, this enrichment of GZMB+Bregs in plasmablasts is not only an effect of the B cell stimulation by BCR and CD40L as these cells display this terminally differentiated profile *in vivo* (17, 40). This is further supported by the GZMB+Bregs transcriptional profile associated proliferation, cell cycle and metabolism as well as the downregulation of genes linked to B cell activation and antigen presentation.

We previously reported on differentially expressed genes between GZMB+Bregs and non-Bregs using bulk RNAseq in a meta-analysis study (41). Intersection of these two datasets identified 24 common genes, which were mainly associated to activation. Similarly, the 149 genes specific to GZMB+Bregs found in this study were compared with IL-10+Bregs signatures reported in the literature (34–36) but with only 6% overlap of genes mainly associated with proliferation, which is concordant with the low level of IL10+ B cells in GZMB+Bregs. These data further underline the extensive Breg heterogeneity and the absence of common signatures between these different Breg subsets (42).

GZMB+Bregs have been shown by us and other to prevent both T cell proliferation and effector functions (14, 17, 39, 40). In accordance, several effector genes such as *GZMB*, *IFI6*, *MX1* were found downregulated at a transcriptomic level in CD4+CD25- T cells cocultured with GZMB+Bregs. Similarly, CD4+CD25- T cells cocultured with GZMB+Bregs displayed a transcriptional profile associated with inhibition of IL-2-STAT5, KRAS, Interferon-α and -γ signaling and also apoptosis pathways whereas these profiles were increased in T cells cocultured with non-Bregs. These data are concordant with the fact that anti-CD3/anti-CD28 stimulation does induce proinflammatory cytokine secretion, KRAS and Activation-induced Cell Death (AICD) (43) in T cells, and are prevented by GZMB+Bregs.

The exact mechanisms by which GZMB+Bregs suppress effector T cell response are still unknown. As previously shown, only a restricted proportion of GZMB+Bregs express IL-10, in accordance with the fact that blocking IL-10 does not suppress their function (16). Similarly, we also found here that TGF-β was upregulated in GZMB+Bregs but only in a very small number of GZMB+Bregs and blocking TGF-β did not suppress their suppressive function on T cells (14). Using a prediction model to analyze the interactions between ligands present in GZMB+Bregs and their receptors on T cells, we were able to identify 5 ligands expressed by B cells, LTA, HMGB2, GAL3, MIF and GZMB. Except for GZMB, none of these molecules, until yet, were reported to be involved in B cell regulatory functions independently of the regulatory B cell subtypes. Both LTA, a tumor necrosis factor (TNF) superfamily member, and the GAL3 lectin were then validated to be higher expressed at the transcript and protein levels in GZMB+Bregs, as compared to non-Breg cells and activated B cells. This highlights their potential usefulness as specific biomarkers to detect and isolate GZMB+Bregs.

Interestingly, we found that LTA inhibition blocked the induction of GZMB in GZMB+Bregs but also restored T cell proliferation in a dose dependent manner, showing that, in contrast to GAL3, LTA is not only a biomarker for GZMB+Bregs but also involved in their function. LTA is a 22kDa cytokine expressed by lymphoid cells upon activation (44, 45). When produced as a soluble homotrimer form, LTA has two receptors TNFR1 and TNFR2 (46). LTA can also form a membrane-bound heterotrimer complex with lymphotoxin beta (LTB) to bind with its receptor LTBR (46). In our dataset, the increased expression of LTA but not LTB likely favors the homotrimer form of LTA in GZMB+Bregs. LTA is involved in numerous pathologies, participating in a variety of activities as evidenced *in vitro* and *in vivo* in several studies. As part of the TNF family, LTA has been extensively described as pro-inflammatory (45–50). Yet, such pro-inflammatory cytokines have already shown capacities to induce regulatory B cells (51). Besides, different studies described regulatory functions of LTA. LTA^-/-^ mice have been shown to fail to develop specific tolerance to OVA and are associated with altered development of mesenchymal lymph nodes whereas TNFa^-/-^ and LTB^-/-^ mice also have altered development of mesenchymal lymph nodes and still develop tolerance to OVA (51). Concordant with our results, the adoptive transfer of LTA^-/-^ bone marrow cells induces enhanced Th1 responses compared to bone marrow cells from wild type mice, showing that LTA prevents effector response independently of lymph node formation (52). Yet, TNFR1 and TNFR2 activation on T cells have been implied in NF-kB signaling and mostly associated with effector functions induction. Whereas in LTA^-/-^ mice, T cell activation is impaired, adoptive transfer of LTA^-/-^ splenocytes in WT mice induced normal T cell functions, indicating that LTA implication in T cell effector functions is mainly associated with mesenchymal lymph nodes formation (53). It is thus complex to characterize the direct role of LTA on T cells *in vivo* . However, in the literature LTA signaling in T cells was mostly associated with NF-kB activation and its involvement in regulatory mechanisms is still unclear. LTA has been shown to be upregulated in regulatory T cells, yet Treg-LTA was associated by an increase of effector T cell migration capacity (54). The molecular mechanisms implied in T cell regulation by GZMB, LTA and other potential Bregs ligands remain to be clarified.

**Figure 7:**
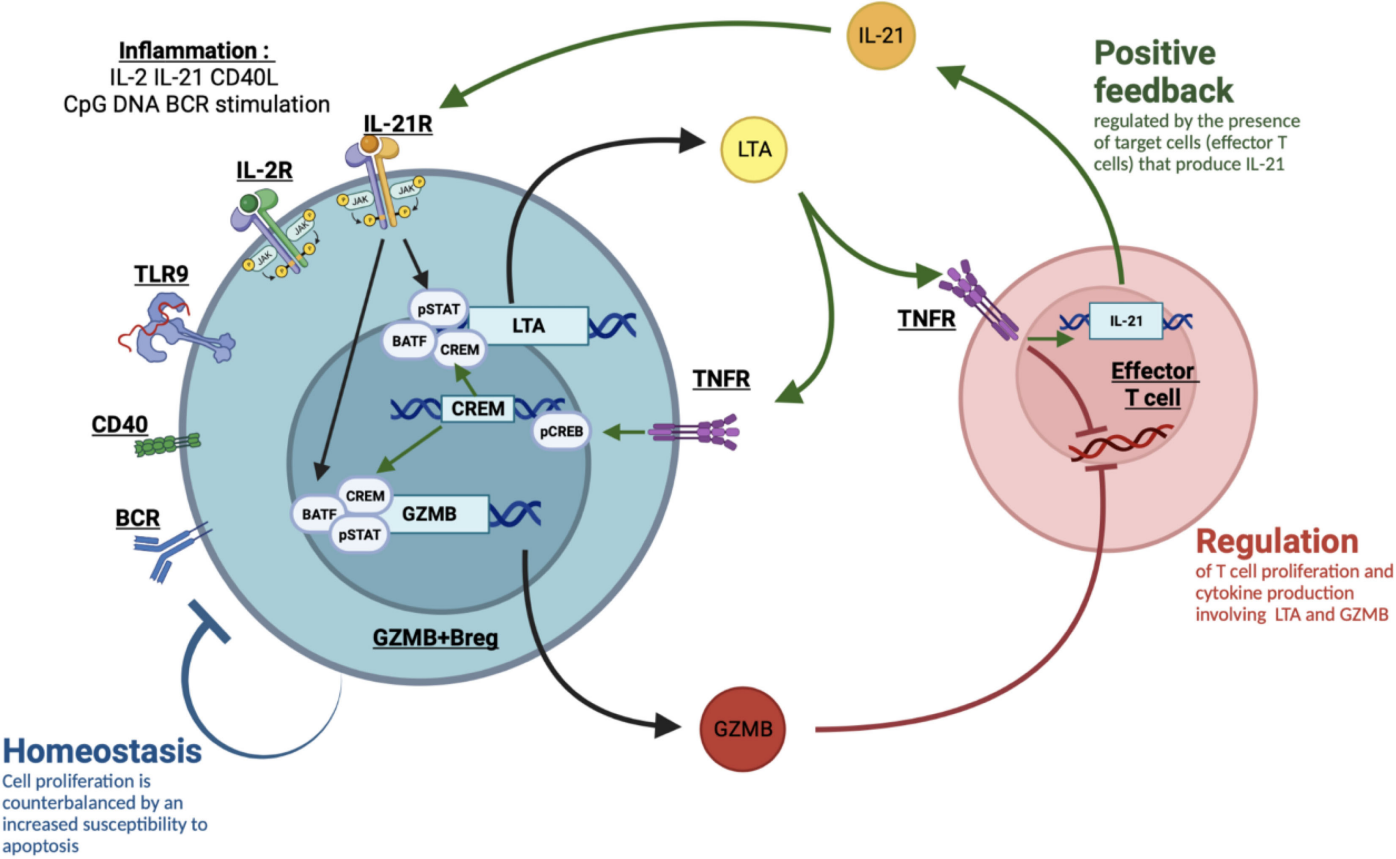
Proposed model of LTA/GZMB interaction in GZMB+Bregs. Upon inflammatory conditions involving IL-2, IL-21, CD40L, CpG DNA and BCR stimuli, B cells differentiate into a GZMB+ regulatory B cell phenotype. IL-21 stimulation is a potent activator of both pSTAT1, pSTAT3 and BATF. Among the targets of pSTATs, we found LTA, that is upregulated in GZMB+Bregs. LTA binding on its receptors TNFR1 and TNFR2 has multiples effects. First, it directly inhibits T cell proliferation. It also increases IL-21 production in T cells thus involving positive feedback on the GZMB+Bregs that express higher level of IL21R. This loop requires the presence and the functional activity of the target T cells to produce IL-21. Last, in the GZMB+Bregs, LTA binding on TNFR activates CREM transcription through CREB phosphorylation. CREM is a transcription factor associated to the promoters of LTA and GZMB, thus associated with LTA and GZMB expression on the GZMB+Bregs. GZMB is also a direct target of BATF. This leads to the suppressive properties of GZMB+Bregs through GZMB and LTA. To counterbalance the positive feedback, GZMB+Bregs have increased apoptosis susceptibility.

We found a correlation between LTA and GZMB expression in GZMB+Bregs and evidenced an interdependency of these two molecules, with LTA being important in the induction of GZMB+Bregs cells and in their suppressive properties. By cross-comparing the lymphotoxin KEGG pathways with the transcription factors database genehancer (31), we found that the transcription factor CREM, upregulated in GZMB+Bregs can regulate the transcription of LTA and GZMB. CREM expression in T cells has been associated with TNFRs activation (55, 56) and the induction of IL-21 production (57). We have previously shown that B cells from tolerant patients increased IL-21 expression by T cells but not B cells from stable patients and HVs (14). As this cytokine appears instrumental for GZMB+Bregs proliferation (16), this suggests a feedback loop in GZMB+Bregs through LTA binding on the T cell and IL-21. Altogether, these data reinforce the potential link between LTA and GZMB in GZMB+Bregs, as illustrated in our proposed model (**Figure 7**). Upon inflammatory conditions involving IL-2, IL-21, CD40L, CpG DNA and BCR stimuli, B cells differentiate into a GZMB+ regulatory B cell phenotype. IL-21 stimulation is a potent activator of both pSTAT1 and pSTAT3 in the B cells (58). Interestingly, the group of Jahrsdörfer already reported in GZMB+Bregs that IL-21 induced the production of GZMB via a JAK1/3 a mechanism dependent on the activation of pSTAT3 (59). Among the targets of pSTATs, we found LTA, that is upregulated in GZMB+Bregs. LTA binding on its receptors has multiples effects. First, in the GZMB+Bregs, LTA was shown to be an autocrine factor for Epstein-Barr Virus infected B cells proliferation (60). Moreover, LTA binding on TNFR was associated with CREM transcription, as shown by its upregulation in GZMB+Bregs. CREM is a transcription factor associated to the promoters of LTA and GZMB, thus associated with LTA and GZMB expression on the GZMB+Bregs. Second, it participates in the inhibition of T cell proliferation, the inhibition of T cell activation, and the induction of IL-21 production in T cells, thus involving positive feedback on the GZMB+Bregs which express higher level of IL-21R.

Altogether, we report for the first time that LTA is specifically expressed in GZMB+Bregs, which may help to better identify and sort this highly heterogenous B cell subset. More importantly, we show that LTA is involved in the induction as well as the suppression of T cells function via GZMB regulation. The exact mechanism of LTA/GZMB cooperation and to which extent LTA directly participate in GZMB+Bregs regulation of effector T cells remain to be further studied. It is likely that LTA may bind to TNFRs on T cells and induce their inhibition (45, 61).

## Data availability statement

The datasets presented in this study can be found in online repositories. The names of the repository/repositories and accession number(s) can be found below: GSE224461 (GEO).

## Ethics statement

The studies involving human participants were reviewed and approved by Ministère de l’Enseignement Supérieur et de la Recherche (MESR) and Comité de Protection des Personnes (CPP). The patients/participants provided their written informed consent to participate in this study.

## Author contributions

SB and NS designed the study. NS, AD, HM, GT, CF and MB carried out the experiments. NS, SB, JP, ND, RD analyzed the data. ND, SB, JP, RD, MG and J-MR drafted and revised the paper. All authors approved the final version of the manuscript.
