# Supplementary material for: Human granzyme B regulatory B cells prevent effector CD4+CD25- T cell proliferation through a mechanism dependent from lymphotoxin alpha
Source: Front Immunol. 2023 Jul 31;14:1183714. doi: 10.3389/fimmu.2023.1183714 (PMC10425555; doi:10.3389/fimmu.2023.1183714)
Supplement: Supplementary file 1 [file DataSheet_1.pdf]

Supplementary Material

Human regulatory B cells prevent effector CD4+CD25- T cell proliferation through a mechanism dependent from granzyme B and lymphotoxin alpha

Nicolas Sailliet[1], Hoa Mai[1], Amandine Dupuy[1], Gaëlle Tilly[1], Cynthia Fourgeux[1], Martin Braud[1], Magali Giral[1], Jean-Michel Robert[2], Nicolas Degauque[1], Richard Danger[1*], Jeremie Poschmann[1*] and Sophie Brouard[1*]

[1] CHU Nantes, Nantes Université, INSERM, Center for Research in Transplantation and Translational Immunology (CR2TI), UMR 1064, ITUN, Nantes, France

[2] Institut de Recherche en Santé 2, Cibles et Médicaments des Infections et de l'Immunité IICiMed-UR1155, Nantes Université, 22, Boulevard Bénoni-Goullin, F-44000 Nantes, France

*** Correspondence:**
Dr. Sophie Brouard, Dr. Jérémie Poschmann, Dr. Richard Danger ; Centre de Recherche en Transplantation et Immunologie INSERM UMR1064, Université de Nantes, Centre Hospitalier Universitaire de Nantes, 30 bd Jean Monnet, 44093 Nantes, France. Phone: +33 2 40 08 74 10, Email: richard.danger@univ-nantes.fr; jeremie.poschmann@univ-nantes.fr; sophie.brouard@univ-nantes.fr

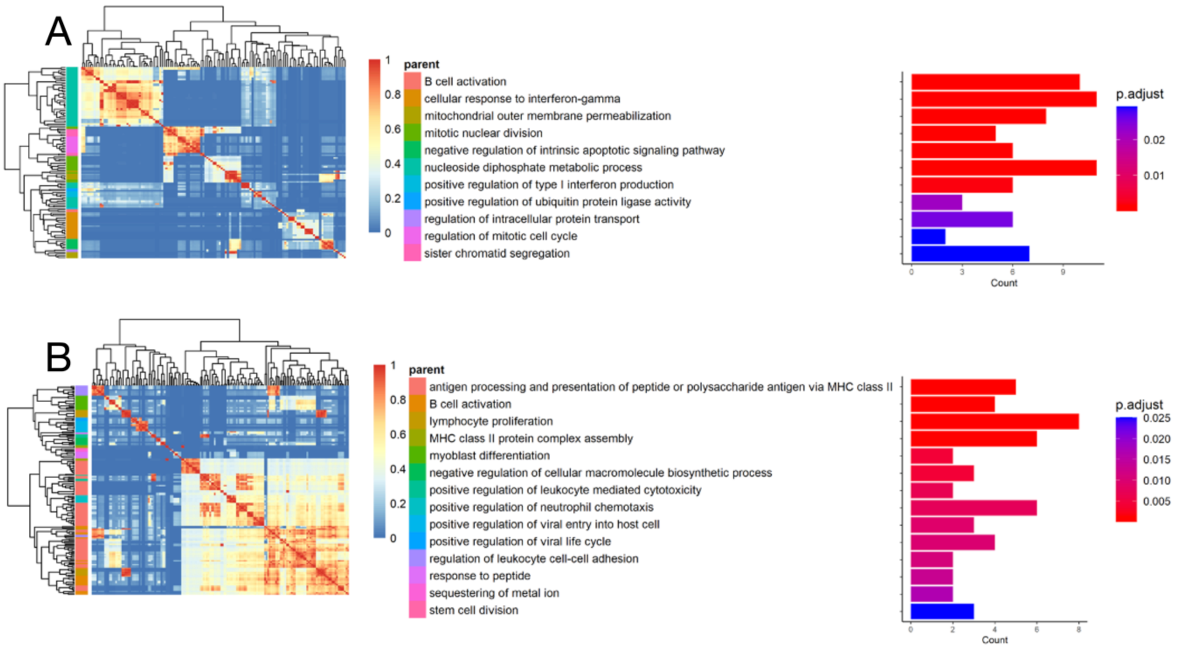

Figure S1: GZMB+Bregs gene ontology reduction: Gene ontology analysis was performed on the 104 up- and 45 down-regulated DEGs. The list of terms was then reduced using the rrvgo package (1.8.0). Heatmaps are representing term reduction of gene ontologies associated to genes enriched **(A)** or repressed **(B)** in GZMB+Bregs vs non-Bregs, color scale represents the homology between terms before reduction. Bar plots represent the adjusted p.value and the number of DEG associated to ontologies enriched or repressed in GZMB+Bregs vs non-Bregs. Only gene sets between 20 and 400 genes were analyzed.

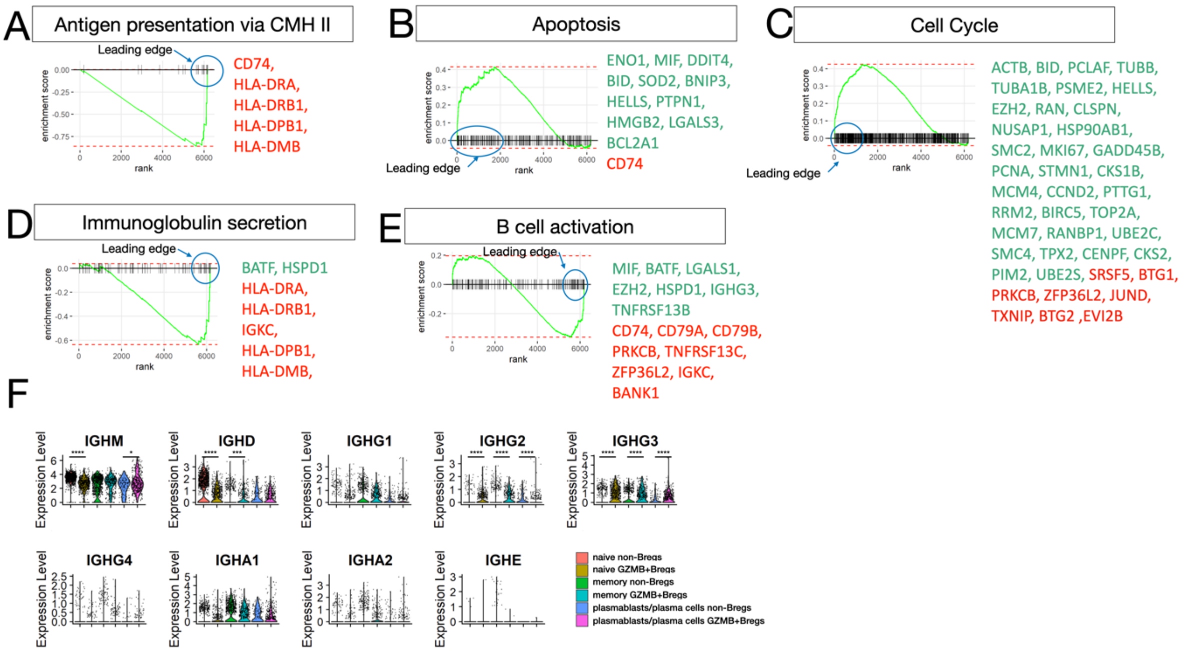

Figure S2: Enrichment analysis of B cell functions and transcriptomic regulators: (A-E) Graphs of GSEA enrichment with highly enriched (green) or repressed (red) contributing genes. (F) Differential expression of Ig heavy chains in Bregs versus non-Bregs.

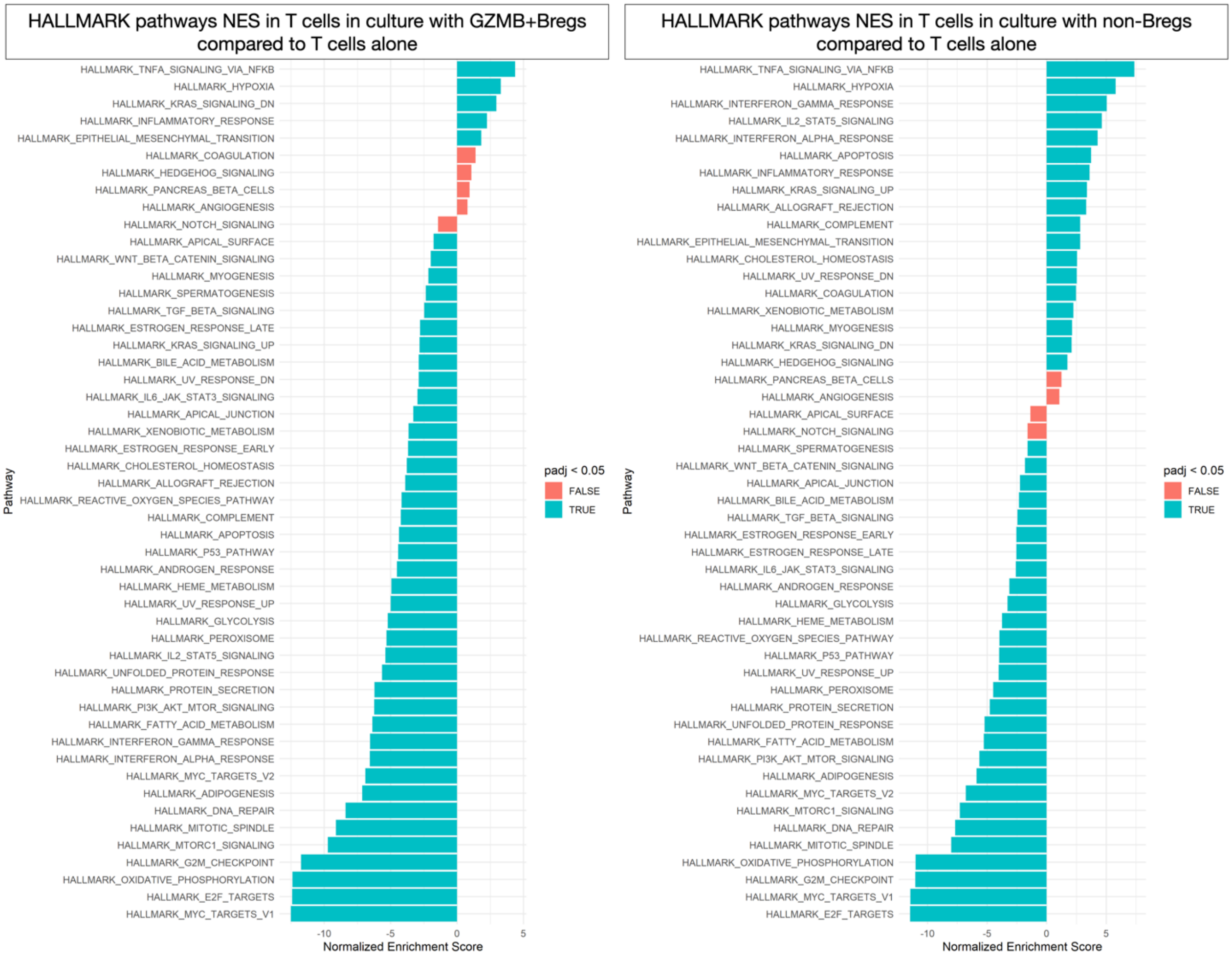

Figure S3: T cell differential expression by GSEA: GSEA analysis performed with the Hallmark gene sets (H) from the Molecular Signature Database (MSigDB) in T cells between T cells in coculture with GZMB+Bregs vs T cells alone (left) and in coculture with non-Bregs vs T cells alone (right) represented as a Barplot with the Normalized enrichment score in lenght. Gene sets with significant adjusted p.values are colored in blue.

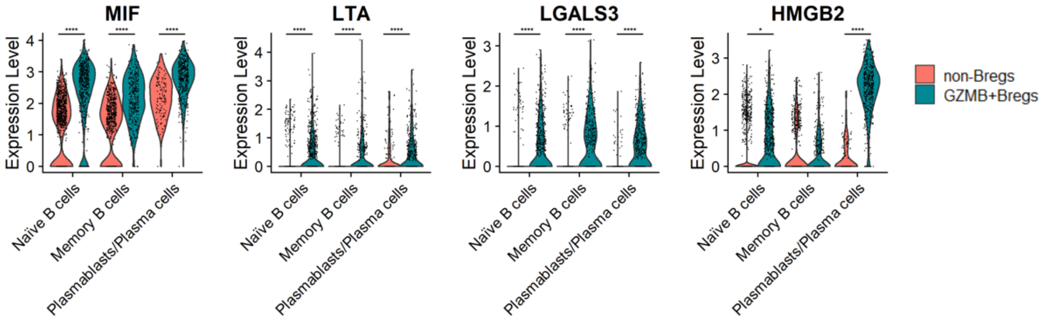

Figure S4: Ligands expression among B cell population: HMGB2, LTA, LGALS3 and MIF expression were compared between non-Bregs and GZMB+Bregs in plasmablasts, naive and memory B cells. Differences were defined as statistically significant when P<0.05 (*), P<0.01 (**), P<0.001 (***) and P<0.0001 (****).

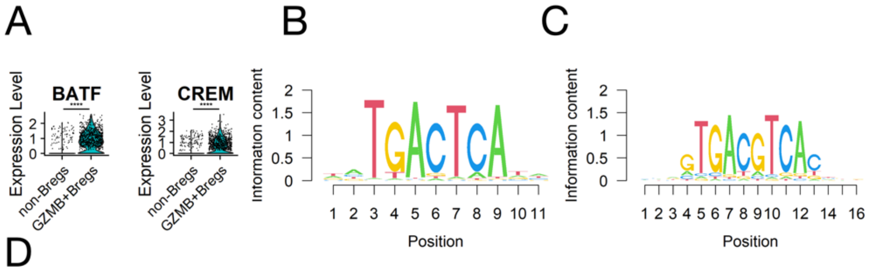

D

Promoter	start	end	score	strand	attributes		
GZMB	-981	-971	1.937211	+	TF=BATF3	class=Basic leucine zipper factors (bZIP)	sequence=TGTGAGCCAAA
GZMB	-874	-864	3.791855	+	TF=BATF3	class=Basic leucine zipper factors (bZIP)	sequence=TATGAACCATC
GZMB	-566	-556	0.260069	+	TF=BATF3	class=Basic leucine zipper factors (bZIP)	sequence=TCAGACTCTCC
GZMB	-463	-453	7.977206	+	TF=BATF3	class=Basic leucine zipper factors (bZIP)	sequence=GCTGAGTCATG
GZMB	-347	-337	1.410389	+	TF=BATF3	class=Basic leucine zipper factors (bZIP)	sequence=CCTGAGTCTGT
GZMB	-15	-5	9.553728	+	TF=BATF3	class=Basic leucine zipper factors (bZIP)	sequence=TTTTACTCAGC
GZMB	-575	-560	3.735123	+	TF=CREM	class=Basic leucine zipper factors (bZIP)	sequence=TGTCTGAGCTCAGACT
GZMB	-418	-403	3.852315	+	TF=CREM	class=Basic leucine zipper factors (bZIP)	sequence=TCAAAGGCGTCATGCA
GZMB	-384	-369	6.739133	+	TF=CREM	class=Basic leucine zipper factors (bZIP)	sequence=GGGCTGATCTCATTCC
GZMB	-226	-211	5.323679	+	TF=CREM	class=Basic leucine zipper factors (bZIP)	sequence=TTCATGACCTCCCCCA
LTA	-663	-653	1.3737722	+	TF=BATF3	class=Basic leucine zipper factors (bZIP)	sequence=TGTTGCTCAAG
LTA	-539	-529	1.7467907	+	TF=BATF3	class=Basic leucine zipper factors (bZIP)	sequence=TCTGGATCAGA
LTA	-181	-171	0.1369543	+	TF=BATF3	class=Basic leucine zipper factors (bZIP)	sequence=GCTGAGCCACC
LTA	-150	-140	4.5758090	+	TF=BATF3	class=Basic leucine zipper factors (bZIP)	sequence=GCTGCCTCACC
LTA	-86	-76	-0.3277754	+	TF=BATF3	class=Basic leucine zipper factors (bZIP)	sequence=GGTGCCACACA
LTA	-357	-342	5.3975040	+	TF=CREM	class=Basic leucine zipper factors (bZIP)	sequence=TCTGGGAGGTCAGGTG
LTA	-333	-318	5.8164256	+	TF=CREM	class=Basic leucine zipper factors (bZIP)	sequence=CCAATGAGGTGAGCAG
LTA	-252	-237	4.9074436	+	TF=CREM	class=Basic leucine zipper factors (bZIP)	sequence=GGTGTGAGGCCAACAC
LTA	-91	-76	4.1813235	+	TF=CREM	class=Basic leucine zipper factors (bZIP)	sequence=AGGGTGGTGCCACACA
LTA	-54	-39	7.3138077	+	TF=CREM	class=Basic leucine zipper factors (bZIP)	sequence=CAGGTGGTGTCATGGG

Figure S5: sequence and sites of fixation for BATF and CREM: (A) Violin plots for the two transcription factors BATF and CREM. **(B-C)** The consensus binding sequences for BATF **(B)** and CREM **(C)**. Size of the letters are corresponding to the probability of getting the given nucleotide at that position. **(D)** Promoter sequences (0 to -1000 pb) of LTA and GZMB were fetched and scanned using the TFBSTools package to highlight patterns of BATF and CREM with a threshold of 70% of homology with their binding sequences. Differences were defined as statistically significant when P<0.05 (*), P<0.01 (**), P<0.001 (***) and P<0.0001 (****).
